# Remarkably enhanced second harmonic generation in Janus HfXY (X, Y = O, S, Se, Te; X ≠ Y) monolayers

**DOI:** 10.3389/fchem.2026.1986563

**Published:** 2026-10-26

**Authors:** Shi-Qi Li, Zhijie Lei, Ruizhao LI, Yunxiao Ban, Xiaozhendong Bao, Jinghong Ding, Hanlin Su, Anbing Zhang, Hongfei Yin

**Affiliations:** 1 School of Physics and Electronic Engineering, Jiangsu University, Zhenjiang, Jiangsu, China; 2 Quantum Sensing and Agricultural Intelligence Detection Engineering Center of Jiangsu Province, Zhenjiang, China; 3 School of Physics and Electronic Information Engineering, Ningxia Normal University, Guyuan, Ningxia, China; 4 Key Laboratory of Materials Modification by Laser, Ion and Electron Beams (Dalian University of Technology), Ministry of Education, School of Physics, Dalian, China

**Keywords:** electronic property, first-principle calculation, janus monolayers, second harmonic generation, tetragonal phase

## Abstract

Designing new two-dimensional (2D) materials with enhanced second harmonic generation (SHG) and uncovering the underlying mechanisms is crucial for enabling high-performance nonlinear photonics. In this work, we design a new Janus structure HfXY (X, Y = O, S, Se, Te) with tetrahedral phase and investigate its structural stability, electronic properties, and SHG responses using first-principles calculations. Phonon spectrum analysis confirms that three of these monolayers, HfOSe, HfOTe, and HfSTe, are dynamically stable semiconductors. All three dynamically stable Janus monolayers are indirect semiconductors with valence band maxima located at the Γ point and conduction band minima at the S point. HfOSe and HfSTe monolayers exhibit pronounced out-of-plane SHG with nonlinear susceptibility components reaching the order of 10^2^ pm/V, 1–2 orders of magnitude higher than in conventional bulk nonlinear optical crystals, and the charge asymmetry also enhances the SHG response of HfOSe and HfSTe beyond that of their pristine counterparts. By uncovering the intrinsic origins of their outstanding SHG activity, our findings position tetragonal Janus monolayers as a promising materials platform, stimulating the exploration of multiphase Janus systems and their implementation in cutting-edge nonlinear photonic devices.

## 1 Introduction

As a prototypical second-order nonlinear optical (NLO) phenomenon, second harmonic generation (SHG) plays a pivotal role in modern photonics (Boyd et al., 2008; Shen, 1984; Huang et al., 2024; Autere et al., 2018; Khan et al., 2021), enabling diverse applications in pulsed lasers (Keller, 2003), electro-optical modulators (Wooten et al., 2000; Elshaari et al., 2018; Kim et al., 2008), all-optical switches (Li, 2017; Chang et al., 2014; Champagne et al., 2012), and frequency conversion devices (Sun et al., 2004; Sun et al., 2007; Bo et al., 2006). In addition to photonic applications, SHG has demonstrated notable utility in molecular detection (Asadipour et al., 2024), biosensing, and food quality analysis (Watanabe et al., 2025; Brüggemann et al., 2010), owing to its heightened chemical selectivity and adaptability compared with conventional NLO techniques (e.g., surface-enhanced Raman spectroscopy, SERS) (Sun et al., 2021; Zhai et al., 2021; Chen et al., 2022). Since the experimental realization of graphene, low-dimensional materials have attracted extensive attention due to their unique optical and electronic properties (Pei et al., 2025; Yang et al., 2025; Zhou et al., 2026; Yu et al., 2026; Li et al., 2025). Among them, two-dimensional (2D) materials constitute an ideal platform for SHG, as their confined electronic states and reduced electric screening yield strong nonlinear susceptibilities (Huang et al., 2024; Autere et al., 2018; Khan et al., 2021), while their atomically thin geometry ensures seamless compatibility with on-chip integration and inherently satisfies phase-matching constraints (Zhao et al., 2016). Recent experimental advances have further demonstrated this potential through enhanced SHG in 3R-MoS_2_ metasurfaces (Peng et al., 2025), visible second-harmonic emission from WSe_2_-integrated silicon photonic chips (Shi et al., 2025), and enhanced frequency conversion in MoS_2_-integrated silicon nitride waveguides (Rehan et al., 2026). These developments highlight the prospects of 2D materials for compact nonlinear light sources and on-chip frequency conversion.

As a prominent class of 2D materials, transition metal dichalcogenides (TMDs) (Manzeli et al., 2017; Choi et al., 2017; Kolobov and Tominaga, 2016; Wang et al., 2012; Strasser et al., 2022; Wei et al., 2019; Friedman et al., 2017) possess unique structural and optical characteristics that endow them with distinct advantages for NLO applications (Strasser et al., 2022; Wei et al., 2019). TMDs crystallize in multiple polymorphs, including the semiconducting 2H (Friedman et al., 2017), the metallic 1T (Friedman et al., 2017), and the semimetallic 1T’ (Tan et al., 2017) phases, each exhibiting markedly different electronic and optical responses suitable for SHG related technologies. Recently, the structural feasibility of a tetrahedral phase in TiX_2_ (X = S, Se, Te) has been predicted via first-principles calculations, expanding the landscape of 2D TMD polymorphs (Chen et al., 2025). However, despite the substantial progress enabled by existing polymorphs, their NLO performance remains fundamentally constrained. In addition, the pronounced out-of-plane symmetry together with the absence of a built-in vertical electric field suppresses the out-of-plane nonlinear polarization in monolayer TMDs, which are increasingly important for device-level implementation.

Constructing Janus architectures has emerged as a promising strategy for engineering the structural, electronic, and optical properties of two-dimensional materials (Wang et al., 2025; Li et al., 2022; Pang et al., 2021; Li et al., 2018; Tang and Kou, 2022). Compared with pristine TMD monolayers with equivalent chalcogen layers, Janus structures possess chemically distinct top and bottom chalcogen layers, resulting in an asymmetric atomic configuration, reduced crystal symmetry, and an out-of-plane dipole and built-in electric field (Li et al., 2018; Tang and Kou, 2022; Lu et al., 2017; Zhang et al., 2017; Li et al., 2019). These structural and electrostatic characteristics can modify the band gaps, band-edge positions, charge distribution, and optical properties of Janus TMDs (Pang et al., 2021; Li et al., 2018; Tang and Kou, 2022; Lu et al., 2017; Zhang et al., 2017; Li et al., 2019; Van Thanh et al., 2020; Aqsa et al., 2022; Kushal et al., 2025; Liu et al., 2024). Janus structures have been theoretically predicted and experimentally realized in 2H, 1T, and 1T’ TMDs (Lu et al., 2017; Zhang et al., 2017; Li et al., 2019; Van Thanh et al., 2020; Sant et al., 2020; Lin et al., 2020; Aqsa et al., 2022; Kushal et al., 2025; Liu et al., 2024; Shi et al., 2023; Zhao et al., 2025), where their reduced symmetry and polarity can also give rise to distinctive second-order nonlinear optical responses, including out-of-plane SHG (Wang et al., 2025; Li et al., 2022; Pang et al., 2021). However, previous studies have mainly focused on hexagonal and distorted TMD systems, while systematic investigations of tetragonal-phase Janus HfXY monolayers remain limited, particularly regarding the relationship among symmetry reduction, built-in electric fields, electronic structures, and SHG responses.

In this work, we construct six Janus structures of tetragonal-phase TMDs HfXY (X, Y = O, S, Se, Te, and X ≠ Y) and investigate their structural stability, electronic properties, and SHG response. Phonon dispersion calculations establish that the Janus monolayers HfOSe, HfOTe, and HfSTe possess dynamic stability. Their intrinsic vertical asymmetry and built-in electric field furnish an effective and controllable platform for enhancing and modulating the second order nonlinear optical response. HfOSe and HfSTe Janus monolayers exhibit remarkably enhanced out-of-plane SHG, featuring five independent SHG susceptibility tensor components with values approaching 10^2^ pm/V, 1–2 orders of magnitude higher than conventional bulk materials and significantly surpassing their pristine counterparts HfX_2_. Momentum-space analysis of monolayer HfOSe and HfSTe reveals that their enhanced SHG responses are strongly localized near high-symmetry points (Γ for HfOSe and S for HfSTe), with each tensor component dominated by specific electronic transitions.

## 2 Calculation methods

All calculations were carried out with the Vienna Ab initio Simulation Package (VASP) (Kresse and Furthmüller, 1996a; Kresse and Furthmüller, 1996b) within the framework of density functional theory (DFT). The exchange–correlation effects were described using the Perdew-Burke-Ernzerhof (PBE) functional under the generalized gradient approximation (GGA) (Perdew et al., 1996; Troullier and Martins, 1991) with the plane-wave cutoff of 500 eV. The coupling between atomic cores and valence electrons was described using the projector-augmented wave (PAW) approach (Blöchl, 1994). Structural optimization was performed until the residual force on each atom fell below 0.01 eV/Å, while the total energy was converged to within 1 × 10^−6^ eV. A vacuum spacing of 18 Å was introduced to eliminate spurious interactions among periodic images. Phonon dispersions were computed using the finite-displacement approach implemented in PHONOPY (Gonze et al., 1994; Giannozzi et al., 1991). To compensate for the band gap underestimation of the PBE functional, electronic structures were further evaluated using the HSE06 hybrid functional (Heyd et al., 2003).

Aversa and Sipe first established the length-gauge formalism for evaluating second-order nonlinear susceptibilities (Aversa and Sipe, 1995), which was subsequently extended by Rashkeev to account for all symmetry-allowed contributions (Rashkeev et al., 1998). The second-order nonlinear susceptibility $\chi_{abc}^{\left(2\right)}\left(-2\omega ,\omega ,\omega \right)$ was calculated within the independent particle approximation, incorporating pure interband contributions $\chi_{inter}^{\left(2\right)}\left(-2\omega ,\omega ,\omega \right)$ , intraband transition processes $\chi_{intra}^{\left(2\right)}\left(-2\omega ,\omega ,\omega \right)$ , and the modulation of interband terms by intraband terms $\chi_{\mathrm{mod}}^{\left(2\right)}\left(-2\omega ,\omega ,\omega \right)$ (Sipe and Ghahramani, 1993; Sharma et al., 2003). In addition, a scissor correction, defined as the band-gap difference between PBE and HSE06, was applied to partially remedy the missing many-body effects in the SHG calculations. A 33 × 33 × 1 k-point mesh and a broadening parameter of 0.05 eV were used for the SHG calculations. The total number of bands included was four times the number of valence bands, and convergence with respect to the k-point mesh and the number of bands was verified. Further computational details are provided in Supplementary Section S1 of the Supporting Information.

## 3 Results and discussion

Pristine HfX_2_ adopts a three-atomic-layer X-Z-X configuration with the transition metal Hf sandwiched between two chalcogen layers, forming a tetragonal lattice belonging to the $P\bar{4}m2$ space group (Chen et al., 2025). By replacing the chalcogen atoms on one side of a tetragonal-phase monolayer HfX_2_ (X = S, Se, Te) with different chalcogen species, a tetragonal-phase Janus HfXY (X, Y = O, S, Se, Te, and X ≠ Y) structure is obtained (Figures 1a–c). The introduction of different chalcogen species makes the two chalcogen sublayers crystallographically inequivalent, thereby lowering the crystal symmetry from $P\bar{4}m2$ ( $D_{2d}$ ) in pristine HfX_2_ to Pmm2 ( $C_{2v}$ ) in Janus HfXY. This symmetry reduction reflects the asymmetric chemical environment between the two sides of the Janus monolayer (Figure 1b).

**FIGURE 1:**
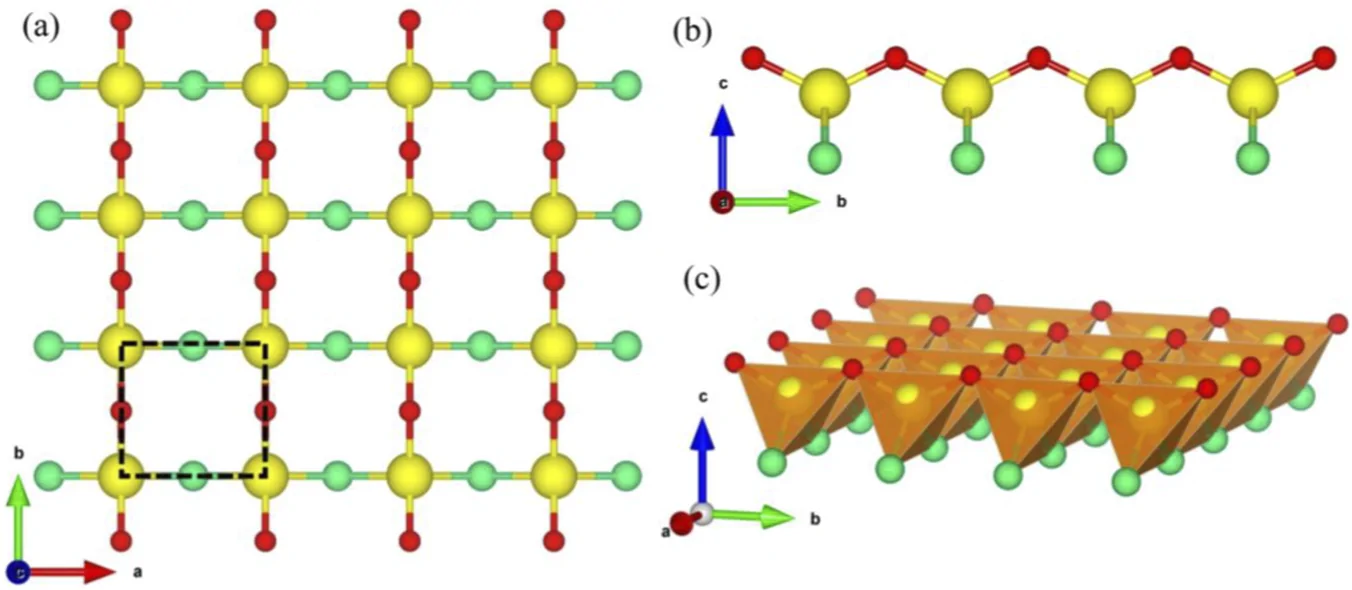
**(a)** Top and **(b)** side views of the geometric structure of the Janus HfXY monolayers. **(c)** Corresponding tetrahedral coordination configuration. The yellow sphere denotes the Hf atom, while the red and green spheres represent the two distinct chalcogen atoms.

After full structural relaxation using the PBE functional, the optimized structural parameters are summarized in Table 1. Across all six Janus HfXY monolayers, the in-plane lattice parameters *a* and *b* systematically increase with the atomic radius of the constituent chalcogen atoms. Among them, HfSeTe exhibits the largest lattice constants (*a* = 4.20 Å and *b* = 4.08 Å). This monolayer also shows the longest Hf-X and Hf-Y bond lengths and the greatest vertical separation between the two outer chalcogen layers, attributable to the relatively large atomic radius of Se and Te. Besides, the lattice parameters *a* and *b*, as well as the Hf-X and Hf-Y bond lengths, are closely related to the difference in electronegativity between the two chalcogen atoms in the outer layers. When the electronegativities of the top and bottom chalcogens are similar, *a* and *b* remain nearly identical, and the Hf-X and Hf-Y bonds are relatively uniform. In contrast, a larger electronegativity difference between the layers leads to more pronounced disparities in *a* and *b*, a further reduction in crystal symmetry, and more significant bond length variations. Taking HfOTe as an example, the maximal electronegativity difference between O and Te induces the largest disparities in lattice parameters *a* and *b* as well as in Hf-X and Hf-Y bond lengths.

**TABLE 1:** The calculated lattice constant *a* and *b*, bond length *d* and thickness of the Janus HfXY (X, Y = O, S, Se, Te and X ≠ Y) monolayers.

System	*a*(Å)	*b*(Å)	*d*_Hf-*X*_(Å)	*d*_Hf-*Y*_(Å)	Thickness(Å)
HfOS	3.81	3.51	4.30	4.25	2.38
HfOSe	3.92	3.54	4.39	4.35	2.48
HfOTe	4.16	3.61	1.99	2.76	2.64
HfSSe	3.97	3.90	4.64	4.65	2.97
HfSeTe	4.20	4.08	4.91	4.92	3.26
HfSTe	4.18	3.99	2.41	4.84	3.12

Additionally, the dynamical stability of all six Janus HfXY monolayers was examined through phonon-dispersion calculations. Among them, only HfOSe, HfOTe, and HfSTe exhibit phonon spectra free of imaginary frequencies throughout the entire Brillouin zone (Figure 2), confirming their dynamical stability. Each phonon spectrum contains nine vibrational branches comprising three acoustic modes and six optical modes, consistent with the presence of three atoms in the primitive cell. For the three dynamically stable Janus monolayers, all six optical branches remain nondegenerate at the Γ point. Moreover, the optical mode frequencies decrease systematically from HfOSe to HfSTe, a trend dictated by the increasing atomic mass of the chalcogen atoms from O to Te.

**FIGURE 2:**
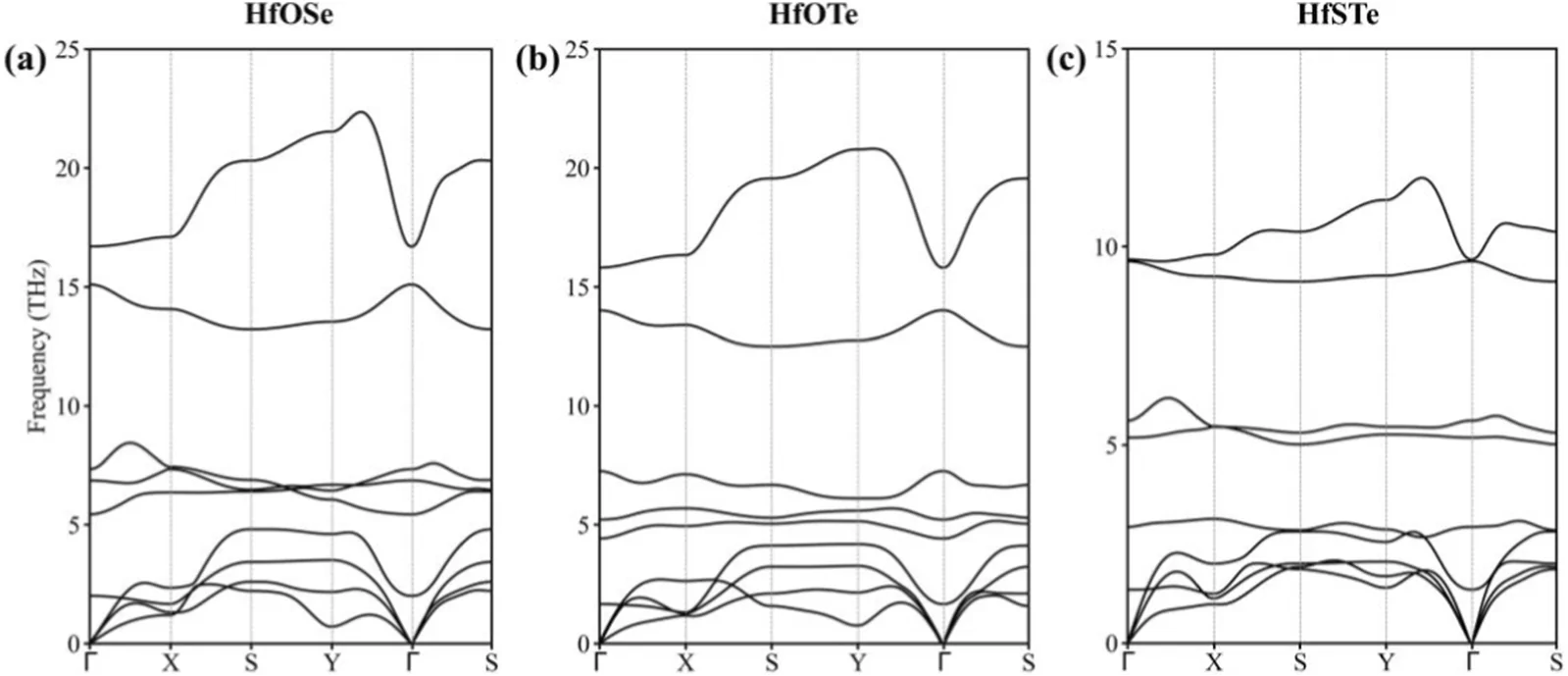
Phonon band structures of **(a)** HfOSe, **(b)** HfOTe and **(c)** HfSTe monolayers, respectively.

Figures 3a–c present the electronic band structures of the dynamically stable monolayer Janus HfXY compounds calculated using both the PBE and the HSE06 hybrid functional. Within the PBE framework, HfOSe and HfSTe are predicted to be semiconductors with bandgaps of 1.05 eV and 0.60 eV, respectively, both exhibiting indirect-gap characteristics (red lines, Figures 3a,c). In contrast, HfOTe shows a valence band maximum that rises above the conduction band minimum (red lines, Figure 3b), leading to a direct overlap between the highest valence and lowest conduction states at the Fermi level and consequently giving rise to metallic behavior. However, given the well-known tendency of the PBE functional to underestimate bandgaps and the relatively modest band overlap observed, employing the HSE06 functional is expected to lift the crossing and restore a finite bandgap. As expected, the HSE06 calculations yield widened bandgaps of 1.85 eV and 1.20 eV for monolayer HfOSe and HfSTe (blue lines, Figures 3a,c), representing increases of approximately 0.5–0.8 eV relative to the PBE results. In addition, monolayer HfOTe no longer exhibits metallic characteristics; instead, it becomes an indirect semiconductor with a bandgap of about 0.5 eV (blue lines, Figure 3b). All three Janus monolayers are thus identified as indirect-gap semiconductors, with the valence band maximum located at the Γ point and the conduction band minimum at the S point (Figures 3a–c). To further clarify the electronic structure modulation induced by Janus engineering, the band structures of pristine HfX_2_ (X = S, Se, Te) monolayers were compared with those of the corresponding Janus HfXY systems (Supplementary Figures S2a–c in the Supporting Information). Both pristine and Janus monolayers exhibit indirect-gap semiconductor characteristics, with identical locations of the valence band maximum at the Γ point. However, replacing one chalcogen layer in pristine HfX_2_ with another chalcogen element introduces an asymmetric chemical environment and significantly modifies the band edge states. Compared with pristine HfX_2_ monolayers, Janus HfXY systems exhibit reduced bandgaps, mainly due to the downward shift of the valence-band states.

**FIGURE 3:**
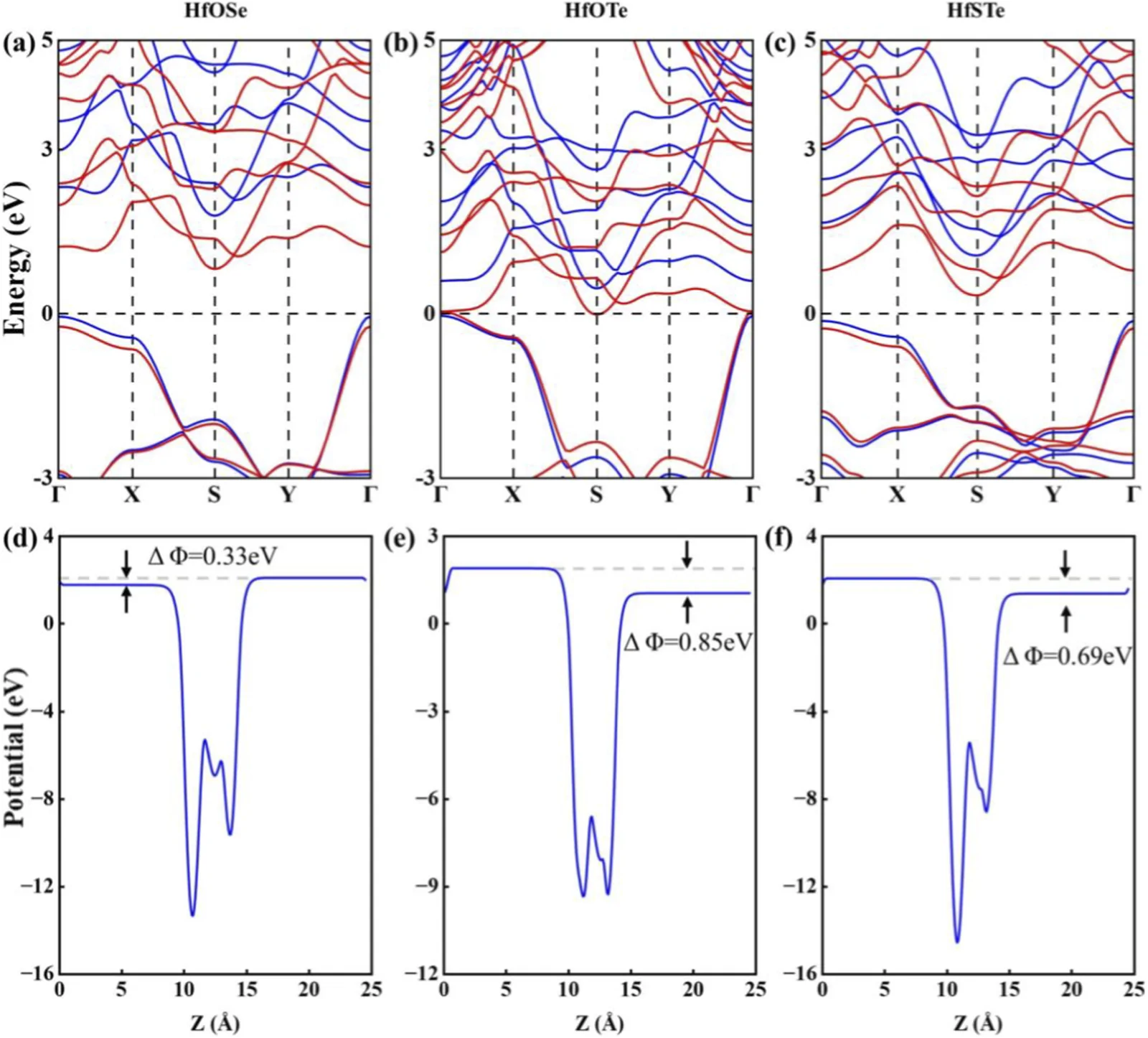
Band structures based on HSE06 (blue lines) and PBE (red lines) functional for **(a)** HfOSe, **(b)** HfOTe and **(c)** HfSTe monolayers. The electrostatic potential energy of **(d)** HfOSe, **(e)** HfOTe and **(f)** HfSTe monolayers along the z direction. The values of work function difference ΔΦ are annotated within the figures.

Due to the intrinsic geometric asymmetry of Janus monolayers, an internal electric field is generated, which can enhance the optical response of the material. Figures 3d–f show the electrostatic potential profiles calculated for three Janus monolayers. The work function characterizes the ability of electrons to escape from the material surface and is defined as the minimum energy required to move an electron from the Fermi level to the vacuum level. A vacuum level offset appears between the two sides of the Janus monolayer, resulting in different work functions for the two distinct surfaces X and Y (Figures 3d–f). In contrast, the corresponding pristine HfX_2_ monolayers exhibit symmetric electrostatic potential profiles without any vacuum-level offset, and therefore show no work-function difference between the two surfaces (Supplementary Figures S2d–f in the Supporting Information). The results indicate that HfOTe exhibits the largest work function difference between its two surfaces, measured at 0.85 eV, with O-side at 5.11 eV and Te-side at 4.26 eV (Figure 3e). In contrast, HfOSe shows the smallest difference of only 0.33 eV, with O-side at 4.89 eV and Se-side at 5.22 eV (Figure 3d).

Based on the band structures, we further investigate the SHG response from the dynamically stable Janus monolayers. Monolayer HfOTe exhibits band overlap within the PBE framework (Figure 3b), although HSE06 removes this artificial overlap and confirms its semiconducting nature, the scissors operator cannot correct band folding that has already occurred. Consequently, we further investigate the SHG responses of HfOSe and HfSTe monolayers by comparing them with their pristine HfX_2_ counterparts. Pristine HfX_2_ adopts a tetragonal lattice characterized by the $P\bar{4}m2$ space group and $D_{2d}$ point-group symmetry. As a noncentrosymmetric structure, pristine HfX_2_ intrinsically permits SHG responses. Its second-order nonlinear susceptibility tensor contains four nonzero components, namely $\chi_{xxz}^{\left(2\right)}$ , $\chi_{yyz}^{\left(2\right)}$ , $\chi_{zxx}^{\left(2\right)}$ , and $\chi_{zyy}^{\left(2\right)}$ , which satisfy the symmetry-imposed relations $\chi_{xxz}^{\left(2\right)}=-\chi_{yyz}^{\left(2\right)}$ and $\chi_{zxx}^{\left(2\right)}=-\chi_{zyy}^{\left(2\right)}$ (Supplementary Figure S3 in the Supporting Information). Upon Janus substitution, the two chalcogen layers become crystallographically inequivalent, reducing the crystal symmetry from $P\bar{4}m2$ ( $D_{2d}$ ) to Pmm2 ( $C_{2v}$ ). This symmetry reduction relaxes the constraints on the second-order nonlinear susceptibility tensor and lifts the symmetry relations among the corresponding tensor components (Figures 4a–d). As a result, the existing components can exhibit substantially different magnitudes in the Janus structures, and an additional out-of-plane component, $\chi_{zzz}^{\left(2\right)}$ , becomes allowed (Figures 4e,f).

**FIGURE 4:**
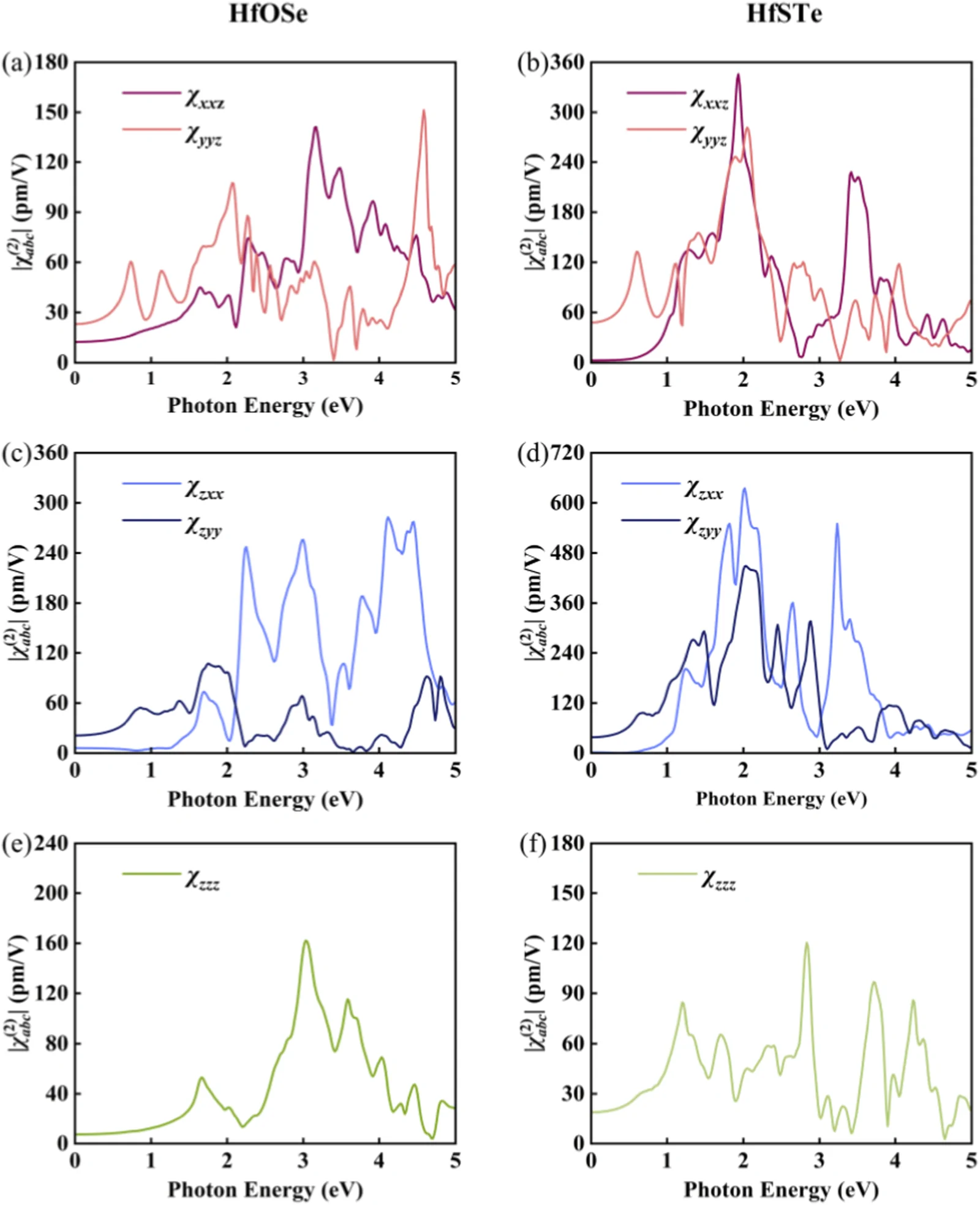
The dispersion of second-order nonlinear susceptibility elements for **(a)**, **(c)**, **(e)** HfOSe and **(b)**, **(d)**, **(f)** HfSTe monolayers, respectively. The *x*, *y*, and *z*-axes denote the Cartesian coordinate axes aligned with the crystallographic directions; the first index of $\chi_{ijk}^{\left(2\right)}$ represents the polarization direction of the generated SHG field, while the second and third indices correspond to the polarization directions of the incident fundamental fields.

The HfOSe and HfSTe monolayers both exhibit remarkably strong SHG responses. First, all their symmetry-allowed $\chi^{\left(2\right)}$ tensor components attain magnitudes approaching 10^2^ pm/V, which is 1–2 orders of magnitude larger than those of widely used bulk nonlinear optical crystals. The enhanced SHG response is closely associated with the inequivalent chemical environments on the two sides of the Janus monolayers. The incorporation of distinct chalcogen species breaks the equivalence between the top and bottom sublayers, inducing asymmetric charge redistribution and an out-of-plane built-in electric field, as evidenced by the vacuum-level offsets between the two surfaces (Figures 3d–f). This structural and charge asymmetry modifies the electronic states and the associated optical transitions, thereby contributing to the enhanced second-order nonlinear optical response. Second, the SHG intensity of the Janus HfOSe and HfSTe monolayers surpass that of their pristine counterparts and other representative 2D materials. Their most pronounced peaks arise from the $\chi_{zxx}^{\left(2\right)}$ contribution, reaching 282.87 pm/V in HfOSe and 635.08 pm/V near 2.0 eV in HfSTe (Figures 4c,d). These magnitudes exceed the respective maxima of pristine HfS_2_ (152.77 pm/V), HfSe_2_ (179.55 pm/V), HfTe_2_ (311.33 pm/V) (Chen et al., 2025), WS_2_ (239.05 pm/V), MoS_2_ (290.1 pm/V), MoSe_2_ (232.74 pm/V), MoSSe (178.21 pm/V), and GaSe (99.77 pm/V) (Supplementary Figure S4 in the Supporting Information). The maximum $\chi_{zxx}^{\left(2\right)}$ peak of monolayer HfSTe is even comparable to the $\chi_{zyy}^{\left(2\right)}$ component of monolayer HfS_2_ under +5% tensile strain (Chen et al., 2025).

Elemental substitution also imposes a substantial modulation on the SHG polarizability. For example, the ordering of the tensor components changes markedly: in HfOSe, the sequence follows $\chi_{zxx}^{\left(2\right)}>\chi_{zzz}^{\left(2\right)}>\chi_{xxz}^{\left(2\right)}>\chi_{yyz}^{\left(2\right)}>\chi_{zyy}^{\left(2\right)}$ (Figures 4a,c,e), whereas in HfSTe it becomes $\chi_{zxx}^{\left(2\right)}>\chi_{zyy}^{\left(2\right)}{>\chi }_{xxz}^{\left(2\right)}{{>\chi }_{yyz}^{\left(2\right)}>\chi }_{zzz}^{\left(2\right)}$ (Figures 4b,d,f). In terms of SHG magnitude, the overall second-order susceptibility of HfSTe exceeds that of HfOSe. These enhancements arise from the synergistic effects of a stronger intrinsic electric field and the band-gap reduction.

Beyond their large SHG susceptibilities, tetragonal Janus HfXY monolayers exhibit distinct nonlinear polarization selection rules compared with typical hexagonal Janus monolayers. Unlike Janus MoSSe and WSSe, which possess both purely in-plane and out-of-plane-related SHG tensor components (Wei et al., 2019; Petrić et al., 2023), the purely in-plane components of the second-order susceptibility tensor vanish in HfOSe and HfSTe owing to symmetry constraints, and all nonzero components involve the out-of-plane direction. Consequently, when the driving electric field is entirely confined to the monolayer plane, the induced second-order nonlinear polarization is exclusively oriented along the out-of-plane direction, providing a route to selectively excite out-of-plane nonlinear polarization. Furthermore, the pronounced out-of-plane nonlinear polarization could couple to optical modes with out-of-plane electric-field components, offering potential for nonlinear frequency conversion in vertical photonic structures (Petrić et al., 2023). These distinctive tensor selection rules, combined with the large SHG susceptibilities, highlight the potential of tetragonal Janus HfXY monolayers for polarization-selective nonlinear photonics.

## 4 Conclusion

In this work, six tetragonal monolayer Janus HfXY materials are constructed by substituting atoms on one side of the monolayer HfX_2_ with different species. Following phonon spectrum calculations, three monolayer semiconductors, HfOSe, HfOTe, and HfSTe, are confirmed to be dynamically stable. The three Janus monolayers all exhibit indirect bandgaps, with the valence band maximum located at the Γ point and the conduction band minimum at the S point. The observed variations in work functions between the distinct surfaces of Janus monolayers indicate the existence of a built-in electric field induced by their intrinsic structural asymmetry. Atomic substitution in Janus monolayers lowers the crystal symmetry relative to pristine HfX_2_, reducing the point group from $D_{2d}$ to $C_{2v}$ . Consequently, the SHG susceptibilities exhibit five nonzero out-of-plane tensor components, while all in-plane components vanish due to symmetry constraints. All symmetry-allowed $\chi^{\left(2\right)}$ tensor components reach magnitudes on the order of 10^2^ pm/V, 1–2 orders of magnitude higher than in conventional bulk nonlinear optical crystals, and the charge asymmetry also enhances the SHG response of HfOSe and HfSTe beyond that of their pristine counterparts. Overall, our work elucidates the intrinsic mechanisms underlying SHG enhancement in Janus HfXY monolayers, providing a foundation for the rational design of high-efficiency 2D NLO materials and devices.

## Data Availability

The original contributions presented in the study are included in the article/Supplementary Material, further inquiries can be directed to the corresponding authors.
