# Supplementary material for: Remarkably enhanced second harmonic generation in Janus HfXY (X, Y = O, S, Se, Te; X ≠ Y) monolayers
Source: Front Chem. 2026 Oct 26;14:1986563. doi: 10.3389/fchem.2026.1986563 (PMC13745471; doi:10.3389/fchem.2026.1986563)
Supplement: Supplementary file 1 [file DataSheet1.docx]

**Supplementary Materials**

**Remarkably Enhanced Second Harmonic Generation in Janus HfXY (X, Y = O, S, Se, Te; X ≠ Y) Monolayers**

Shi-Qi LI^1,4*^, Zhijie Lei^1,4^, Ruizhao LI^2^, Yunxiao Ban^1,4^, Xiaozhendong Bao^3^, Jinghong Ding^1,4^, Hanlin Su^1,4^, Anbing Zhang^3*^, Hongfei Yin^1,4*^

1. *School of Physics and Electronic Engineering, Jiangsu University, Zhenjiang, Jiangsu 212013, China.*
2. *School of Physics and Electronic Information Engineering, Ningxia Normal University, Guyuan, Ningxia 756099, China.*
3. *Key Laboratory of Materials Modification by Laser, Ion and Electron Beams (Dalian University of Technology), Ministry of Education, School of Physics, Dalian 116024, China*
4. *Quantum Sensing and Agricultural Intelligence Detection Engineering Center of Jiangsu Province, Zhenjiang 212013, China.*

Corresponding authors: [shiqili@ujs.edu.cn](mailto:shiqili@ujs.edu.cn), [anbing@mail.dlut.edu.cn,](mailto:anbing@mail.dlut.edu.cn,) [yinhf@ujs.edu.cn](mailto:yinhf@ujs.edu.cn,)

S1. Rationale of Second Harmonic Generation (SHG) calculations

Second-harmonic generation occurs when two photons of frequency $\omega$ propagate through a non-centrosymmetric medium and combine to emit a single photon at 2ω. The induced second-order nonlinear polarization $P_{a}\left( 2\omega\right)$ can be described as follows in terms of the incident electric field $E(\omega)$:

$P_{a}\left( 2\omega\right)=\sum_{a,b} \varepsilon_{0}\chi_{abc}^{\left( 2 \right)}E_{a}\left( \omega\right)E_{b}\left( \omega\right)$	(S1)

where $\chi_{abc}^{(2)}$ represents the second-order susceptibility tensor, with subscript *a* denoting the polarization variable of the emitted photon and subscripts *b*, *c* representing the polarization variables of the incident photons. The second-order nonlinear susceptibility $\chi_{abc}^{(2)}(-2\omega,\omega,\omega)$ was calculated based on the independent particle approximation considering pure interband transition contributions $\chi_{inter}^{(2)}(-2\omega,\omega,\omega)$, intraband transition processes $\chi_{intra}^{(2)}(-2\omega,\omega,\omega)$, and the modulation of interband terms by intraband terms $\chi_{mod}^{(2)}(-2\omega,\omega,\omega)$, which can be expressed as^[72]^:

$\chi_{abc}^{\left( 2 \right)}(-2\omega;\omega,\omega)=\chi_{inter}^{(2)}(-2\omega,\omega,\omega)+\chi_{intra}^{(2)}(-2\omega,\omega,\omega)+\chi_{mod}^{(2)}(-2\omega,\omega,\omega)$	(S2)

These three contributions can be described as:

$\chi_{inter}^{ijk}(-2\omega;\omega,\omega)=\frac{e^{3}}{\hbar^{2}}\sum_{nml}^{'} \int\frac{d\mathbf{k}}{{4\pi}^{3}}\frac{r_{nm}^{a}\{r_{ml}^{b}r_{ln}^{c}\}}{(\omega_{ln}-\omega_{ml})}[\frac{{2f}_{nm}}{(\omega_{mn}-2\omega)}+\frac{f_{ml}}{(\omega_{ml}-\omega)}+\frac{f_{ln}}{(\omega_{ln}-\omega)}]$ (S3)

$\chi_{intra}^{ijk}(-2\omega;\omega,\omega)=\frac{e^{3}}{\hbar^{2}}\int\frac{d\mathbf{k}}{{4\pi}^{3}}[\sum_{nml}^{'} \omega_{mn}r_{nm}^{a}\{r_{ml}^{b}r_{ln}^{c}\}\{\frac{f_{nl}}{\omega_{ln}^{2}(\omega_{ln}-\omega)}-\frac{f_{lm}}{\omega_{ml}^{2}(\omega_{ml}-\omega)}\}-8i\sum_{nm}^{'} \frac{f_{nm}r_{nm}^{a}\{\Delta_{mn}^{b}r_{mn}^{c}\}}{\omega_{mn}^{2}(\omega_{mn}-2\omega)}+2\sum_{nml}^{'} \frac{f_{nm}r_{nm}^{a}\{r_{ml}^{b}r_{ln}^{c}\}(\omega_{ml}-\omega_{ln})}{\omega_{mn}^{2}(\omega_{mn}-2\omega)}]$	(S4)

$\chi_{mod}^{ijk}(-2\omega;\omega,\omega)=\frac{e^{3}}{\hbar^{2}}\int\frac{d\mathbf{k}}{{4\pi}^{3}}[\sum_{nml}^{'} \{\frac{f_{nm}}{\omega_{mn}^{2}(\omega_{mn}-\omega)}\}{\{\omega}_{mn}r_{nm}^{a}\{r_{ml}^{b}r_{ln}^{c}\}-\omega_{lm}r_{nl}^{a}\{r_{lm}^{b}r_{mn}^{c}\}\}-i\sum_{nm}^{'} \frac{f_{nm}r_{nm}^{a}\{r_{mn}^{b}\Delta_{mn}^{c}\}}{\omega_{mn}^{2}(\omega_{mn}-2\omega)}$	(S5)

where $\omega_{mn}=\omega_{m}-\omega_{n}$, $f_{nm}=f_{n}-f_{m}$ is the difference of the energy and the Fermi distribution functions between the n_th_ and *m*_th_ bands, respectively, and $\Delta_{mn}^{b}=(p_{nn}^{b}-p_{mm}^{b})/m$ is the difference between the *n*_th_ and *m*_th_ bands in relation to the velocity matrix. The incident pump light frequency ω contains a small imaginary smearing factor δ: ω → ω + iδ, where δ = 0.05 eV in this work to avoid divergence problem.

S2. Calculated band structures and built-in electric fields of pristine HfX₂

**Figure S2.** Band structures based on HSE06 (blue lines) and PBE (red lines) functional for (a) HfSe_2_, (b) HfSe_2_ and (c) HfTe_2_ monolayers. The electrostatic potential energy of (d) HfS_2_, (e) HfSe_2_ and (f) HfTe_2_ monolayers along the z direction. The values of work function difference $\Delta\Phi$ are annotated within the figures.

S3. Calculated second-order nonlinear susceptibility spectra of pristine HfS₂, HfSe₂, and HfTe₂, showing the non-zero tensor components of $\chi_{ijk}^{(2)}$.

**Figure S3.** The x, y, and z axes denote the Cartesian coordinate axes aligned with the crystallographic directions; the first index of $\boldsymbol{\chi}_{\boldsymbol{ijk}}^{(\mathbf{2})}$ represents the polarization direction of the generated SH field, while the second and third indices correspond to the polarization directions of the incident fundamental fields. The dispersion of second-order nonlinear susceptibility elements for (a), (d) HfS_2_, (b), (e) HfSe_2_, and (c), (f) HfTe_2_ monolayers, respectively.

S4. SHG susceptibilities of representative 2D NLO materials.

Figure S4. Calculated SHG susceptibility spectra $\boldsymbol{\chi}_{\boldsymbol{ijk}}^{(\mathbf{2})}$ as a function of photon energy for representative 2D NLO materials: (a) MoS_2_, (b) MoSe_2_, (c) Janus MoSSe, (d) WS_2_, and (e) monolayer GaSe. All optical spectra were evaluated under the same first-principles computational framework to provide a consistent comparison with the Janus HfXY monolayers.
